# Supplementary material for: Coupled pharmacokinetic model unveils drug-drug interactions in plasma concentration
Source: PLoS One. 2026 Feb 10;21(2):e0339052. doi: 10.1371/journal.pone.0339052 (PMC12890151; doi:10.1371/journal.pone.0339052)
Supplement: S1 File — This file contains: S1 Fig. Amount -time curves of drug X under consideration of parameter α12' - gastrointestinal tract compartment. S2 Fig. Amount -time curves of drug X under consideration of parameter α12' - the central compartment. S3 Fig. Amount -time curves of drug X under consideration of parameter α12' - the peripheral compartment. S4 Fig. Amount -time curves of drug X under consideration of parameter α12' - elimination accumulated. S5 Fig. Amount -time curves of drug X under consideration of parameter α21' - gastrointestinal tract compartment. S6 Fig. Amount -time curves of drug X under consideration of parameter α21' - the central compartment. S7 Fig. Amount -time curves of drug X under consideration of parameter α21' - the peripheral compartment. S8 Fig. Amount -time curves of drug X under consideration of parameter α21' - elimination accumulated. S9 Fig. Amount -time curves of drug X under consideration of parameter α10' - gastrointestinal tract compartment. S10 Fig. Amount -time curves of drug X under consideration of parameter α10' - the central compartment. S11 Fig. Amount -time curves of drug X under consideration of parameter α10' - the peripheral compartment. S12 Fig. Amount -time curves of drug X under consideration of parameter α10' - elimination accumulated. S13 Fig. Noise samples and fitting curves in the central compartment over time- α12'=1. S14 Fig. Noise samples and fitting curves in the central compartment over time- α21'=1. S15 Fig. Noise samples and fitting curves in the central compartment over time- α10'=1. S16 Fig.The fitted curve of drug amount in the central compartment over time- Imeglimin alone. S17 Fig.The fitted curve of drug amount in the central compartment over time- Metformin alone. S18 Fig.The fitted curve of drug amount in the central compartment over time- oral co-administration. S1 Table. Interaction term parameters α10' in a coupled pharmacokinetic model for various pharmacokinetic parameters. S2 Table. Interaction ter [file pone.0339052.s001.docx]

**S1 File. Supplementary**

# 1 Numerical Simulations

Based on the parameter data in **Table 1** in the main body of the paper, the effects of other parameters on PK process are conducted via the control variable method. The amount-time curves in each compartment are shown in **S1-S12 Figs**, where the black, red, and blue curves correspond to the parameter under consideration being equal to zero, greater than zero, and less than zero, respectively.


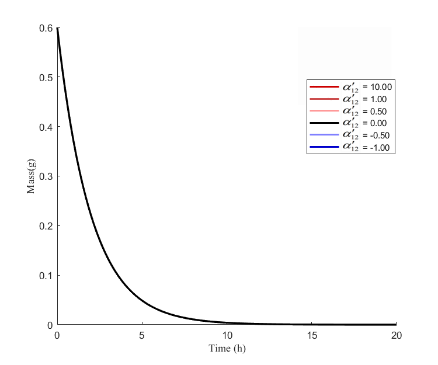


**S1 Fig. Amount -time curves of drug X under consideration of parameter $\alpha'_{12}$- gastrointestinal tract compartment.**


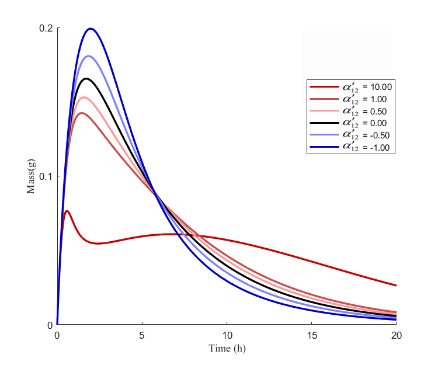


**S2 Fig. Amount -time curves of drug X under consideration of parameter $\alpha'_{12}$- the central compartment.**


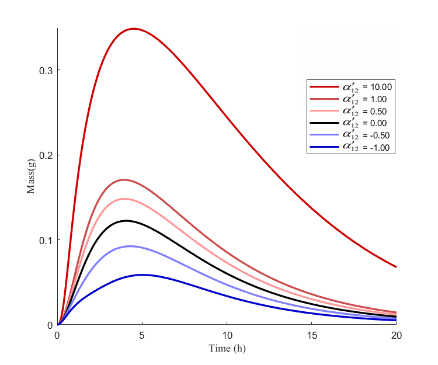


**S3 Fig. Amount -time curves of drug X under consideration of parameter $\alpha'_{12}$- the peripheral compartment.**


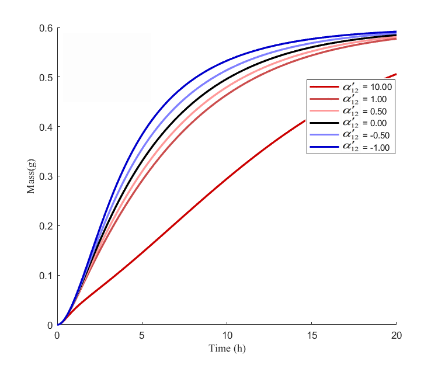


**S4 Fig. Amount -time curves of drug X under consideration of parameter $\alpha'_{12}$- elimination accumulated.**


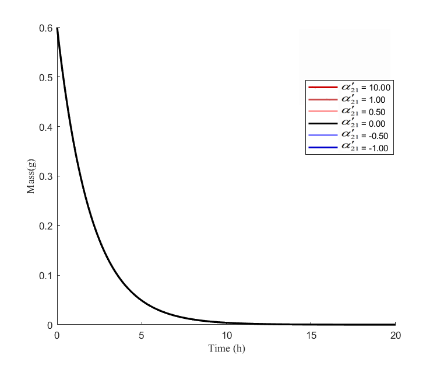


**S5 Fig. Amount -time curves of drug X under consideration of parameter $\alpha'_{21}$- gastrointestinal tract compartment.**


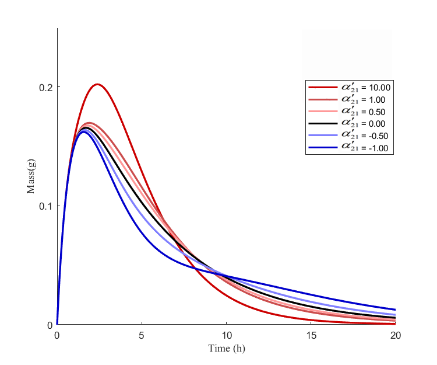


**S6 Fig. Amount -time curves of drug X under consideration of parameter $\alpha'_{21}$- the central compartment.**


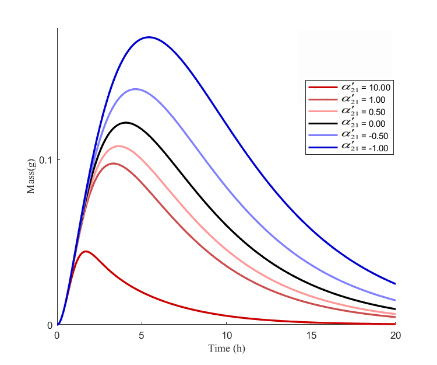


**S7 Fig. Amount -time curves of drug X under consideration of parameter $\alpha'_{21}$- the peripheral compartment.**


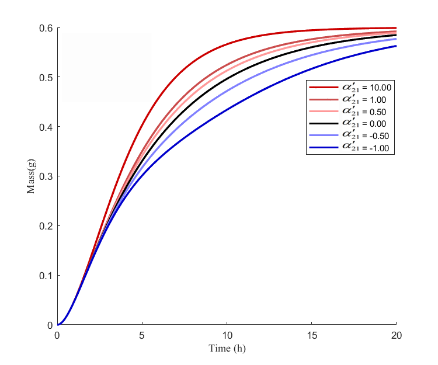


**S8 Fig. Amount -time curves of drug X under consideration of parameter$\alpha'_{21}$- elimination accumulated.**


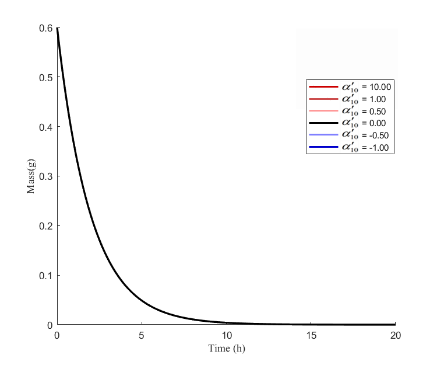


**S9 Fig. Amount -time curves of drug X under consideration of parameter$\alpha'_{10}$- gastrointestinal tract compartment.**


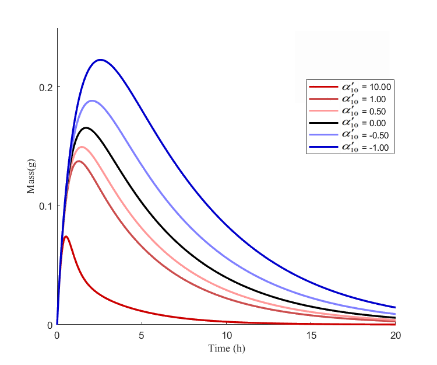


**S10 Fig. Amount -time curves of drug X under consideration of parameter$\alpha'_{10}$- the central compartment.**


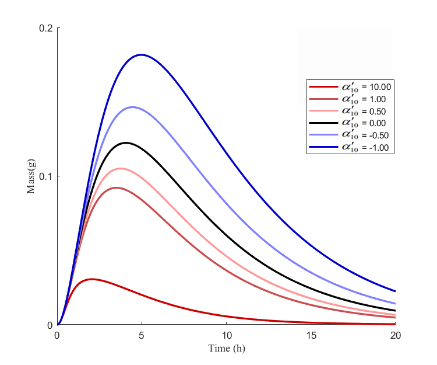


**S11 Fig. Amount -time curves of drug X under consideration of parameter$\alpha'_{10}$- the peripheral compartment.**


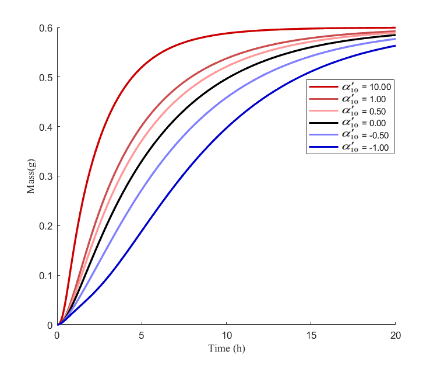


**S12 Fig. Amount -time curves of drug X under consideration of parameter$\alpha'_{10}$- elimination accumulated.**

Based on these intuitive results, the following conclusions can be presented.

（1）It follows from **S1,S5,S9 Figs** that the rate of absorption of drug X from the gastrointestinal tract into the central compartment was not affected with the increase of parameters $\alpha_{12}^{'}$, $\alpha_{21}^{'}$and $\alpha_{10}^{'}$.

（2）As $\alpha_{12}^{'}$, $\alpha_{21}^{'}$and $\alpha_{10}^{'}$ increase, the pre-effect of the smaller amount of drug Y in the central compartment in the early stage has a weaker effect on the transfer of drug X from the central compartment to the peripheral compartment, so that the rate of rise of the red curve in **S2 Fig** in the early stage is similar to that of the black curve. The increase in $\alpha_{12}^{'}$, promotes the entry of drug from the central compartment into the peripheral compartment, and the red curve in the mid-term **S2 Fig** is lower than the black curve. The red curve of late **S2 Fig** is higher than the black curve due to the greater proportion of the drug distributed in the peripheral compartments and being excreted more slowly. The increase in $\alpha_{21}^{'}$ promotes the entry of the drug from the peripheral compartment into the central compartment, and the red curve of the mid-term **S6 Fig** is higher than the black curve. The red curve of late **S6 Fig** is lower than the black curve due to the higher proportion of drug distributed in the central compartment and being excreted faster. The increase in $\alpha_{10}^{'}$ promotes drug excretion from the central compartment, and the red curve in **S10 Fig** is lower than the black curve in the middle and late stages.

（3）The increase of $\alpha_{12}^{'}$ promotes the entry of drugs from the central compartment into the peripheral compartment, and the red curve of **S3 Fig** is higher than the black curve. The increase of $\alpha_{21}^{'}$ promotes the entry of drugs from the peripheral compartment into the central compartment, and the red curve of **S7 Fig** is lower than the black curve. The increase of $\alpha_{10}^{'}$ promotes the excretion of drugs from the body, and the red curve of **S11 Fig** is lower than the black curve.

（4）The increase of $\alpha_{12}^{'}$ promotes the drug in the central compartment to enter the peripheral compartment, the proportion of the drug distributed in the peripheral compartment is larger, and the excretion is slower, the red curve of **S4 Fig** is lower than the black curve. The increase of $\alpha_{21}^{'}$ promotes the drug in the peripheral compartment to enter the central compartment, the proportion of the drug distributed in the central compartment is larger, and the excretion is faster, the red curve of **S8 Fig** is higher than the black curve. The increase of $\alpha_{10}^{'}$ promotes the drug in the central compartment to be excreted, the red curve of **S12 Fig** is higher than the black curve. The red curve in **S12 Fig** is higher than the black curve.

Based on the parameter data presented in **Table 1**, the effects of other interaction parameters on pharmacokinetic indices were analyzed using the control variable method. The impacts of each coupling parameter on the key pharmacokinetic parameters within the coupled PK model are illustrated in **S1-S3 Tables**, respectively.

**S1 Table. Interaction term parameters $\alpha_{10}^{'}$ in a coupled pharmacokinetic model for various pharmacokinetic parameters**

| Drug | Interaction parameters | Pharmacokinetic parameters | | | |
| --- | --- | --- | --- | --- | --- |
|  | $\alpha_{10}^{'}$ (1·103/(mg·h)) | $C_{\max}$ (103·mg) | $T_{\max}$ (h) | AUC  (103·mg·h) | $t_{1/2}$ (h) |
| X | -1 | 0.21094 | 2.3135 | 1.7445 | 7.3324 |
|  | -0.5 | 0.18444 | 1.9904 | 1.4310 | 6.7254 |
|  | 0 | 0.16496 | 1.7292 | 1.1993 | 6.1715 |
|  | 0.5 | 0.15023 | 1.5114 | 1.0241 | 5.6720 |
|  | 1 | 0.13877 | 1.3465 | 0.8886 | 5.2253 |
|  | 10 | 0.07433 | 0.5475 | 0.2440 | 1.9392 |

S2 Table. Interaction term parameters $\alpha_{12}^{'}$ in a coupled pharmacokinetic model for various pharmacokinetic parameters

| Drug | Interaction parameters | Pharmacokinetic parameters | | | |
| --- | --- | --- | --- | --- | --- |
|  | $\alpha_{12}^{'}$ (1·103/(mg·h)) | $C_{\max}$ (103·mg) | $T_{\max}$ (h) | AUC  (103·mg·h) | $t_{1/2}$ (h) |
| X | -1 | 0.19324 | 1.8953 | 1.1995 | 5.3357 |
|  | -0.5 | 0.17801 | 1.8159 | 1.1994 | 5.7197 |
|  | 0 | 0.16496 | 1.7292 | 1.1993 | 6.1715 |
|  | 0.5 | 0.15371 | 1.6268 | 1.1992 | 6.6776 |
|  | 1 | 0.14397 | 1.5191 | 1.1991 | 7.2197 |
|  | 10 | 0.07714 | 0.6104 | 1.1971 | 15.282 |

S3 Table. Interaction term parameters $\alpha_{21}^{'}$ in a coupled pharmacokinetic model for various pharmacokinetic parameters.

| Drug | Interaction parameters | Pharmacokinetic parameters | | | |
| --- | --- | --- | --- | --- | --- |
|  | $\alpha_{21}^{'}$ (1·10^3^/(mg·h)) | $C_{\max}$ (10^3^·mg) | $T_{\max}$ (h) | *AUC*  (10^3^·mg·h) | $t_{1/2}$ (h) |
| X | -1 | 0.16165 | 1.5891 | 1.1988 | 5.2365 |
|  | -0.5 | 0.16324 | 1.6489 | 1.1991 | 5.8362 |
|  | 0 | 0.16496 | 1.7292 | 1.1993 | 6.1715 |
|  | 0.5 | 0.16881 | 1.8051 | 1.1995 | 6.3299 |
|  | 1 | 0.16879 | 1.8839 | 1.1996 | 6.3942 |
|  | 10 | 0.19896 | 2.3677 | 1.1979 | 5.8876 |

Based on these visual results, the following conclusions can be drawn:

1. Trend of $C_{\max}$

As shown in **S1-S3 Tables**, the interaction parameters exhibit distinct influences on the peak concentration $C_{\max}$. When the interaction parameter $\alpha_{10}^{'}$ for elimination from the central compartment increases, $C_{\max}$ decreases significantly, indicating that enhanced clearance reduces the plasma peak concentration. Similarly, when the interaction parameter$\alpha_{12}^{'}$for transfer from the central to peripheral compartment increases, $C_{\max}$ also declines, suggesting that faster distribution to peripheral tissues lowers the concentration in the central compartment. In contrast, an increase in the interaction parameter$\alpha_{21}^{'}$for redistribution from the peripheral to the central compartment raises $C_{\max}$, implying that accelerated return flow increases the central drug level. Overall, these patterns show that enhanced elimination or peripheral distribution reduces $C_{\max}$, whereas stronger backflow from the periphery elevates it.

1. Trend of $T_{\max}$
    As shown in **S1-S3 Tables**, the effects of the three interaction parameters on the peak time $T_{\max}$are generally opposite to those on $C_{\max}$. Increasing $\alpha_{10}^{'}$ shortens $T_{\max}$, reflecting faster elimination and an earlier appearance of the concentration peak. Similarly, increasing $\alpha_{12}^{'}$ also shortens $T_{\max}$, as accelerated peripheral distribution speeds up changes in central concentration. In contrast, a larger $\alpha_{21}^{'}$ delays $T_{\max}$, since stronger redistribution from the periphery slows the rate of central concentration rise, shifting the peak to a later time. Hence, enhancement of clearance or outward distribution accelerates attainment of the steady-state peak, whereas enhanced peripheral backflow delays it.
2. Trend of *AUC*

As shown in **S1-S3 Tables**, the area under the concentration–time curve (*AUC*) decreases as $\alpha_{10}^{'}$ and $\alpha_{12}^{'}$ increase, but slightly rises with increasing $\alpha_{21}^{'}$. Because *AUC* represents total systemic exposure, enhanced interaction parameter for elimination $\alpha_{10}^{'}$or interaction parameter for outward distribution $\alpha_{12}^{'}$ reduces *AUC*, whereas stronger backflow ($\alpha_{21}^{'}$) prolongs circulation and slightly increases *AUC*. These results indicate that *AUC* is jointly regulated by elimination and distribution processes: clearance determines the elimination rate, while distribution governs redistribution and tissue retention.

1. Trend of $t_{1/2}$

As seen in **S1-S3 Tables**, the effects of the three interaction parameters on the elimination half-life $t_{1/2}$ are not uniform, revealing the coupled nature of clearance and distribution in the system.

When $\alpha_{10}^{'}$ increases, $t_{1/2}$decreases markedly, showing that enhanced elimination accelerates drug removal and raises the overall metabolic rate. This trend parallels the decline in *AUC*, confirming the dominant role of clearance in determining systemic residence time.

In contrast, when $\alpha_{12}^{'}$ increases, $t_{1/2}$is prolonged, suggesting that rapid distribution to peripheral compartments slows the decline in central concentration, producing an “apparent extension” of elimination. This reflects a depot-like effect: the drug temporarily stored in peripheral tissues returns slowly, extending the overall half-life.

For $\alpha_{21}^{'}$, the effect on $t_{1/2}$ is nonlinear. At low values, increasing $\alpha_{21}^{'}$ lengthens$t_{1/2}$, but when the backflow rate becomes excessive, $t_{1/2}$slightly decreases. Moderate backflow helps sustain plasma concentration, while excessive return raises the central concentration and triggers faster clearance. This “increase–then–decrease” trend shows that peripheral backflow can maintain systemic levels within limits but accelerates elimination when overly strong.

Overall, the changes in $t_{1/2}$ reveal distinct mechanistic roles of each interaction parameter in determining drug retention time:

- $\alpha_{10}^{'}$ (central→elimination): governs the elimination rate—higher values accelerate clearance and shorten $t_{1/2}$;
- $\alpha_{12}^{'}$ (central→peripheral): produces a delayed-release effect, lengthening $t_{1/2}$;
- $\alpha_{21}^{'}$(peripheral→central): moderately extends $t_{1/2}$ but, when excessive, promotes rapid elimination.

Collectively, these results demonstrate that in the coupled pharmacokinetic model, clearance, distribution, and backflow are not independent linear processes but dynamically interconnected through parameter coupling. The clearance parameter $\alpha_{10}^{'}$ controls the system’s “outflow”, $\alpha_{12}^{'}$ regulates intercompartmental redistribution, and $\alpha_{21}^{'}$ adjusts systemic recirculation efficiency. Their combined effects determine the overall exposure, residence time, and dynamic evolution of drug concentration peaks and troughs in vivo.

# 2 Numerical Examples

Numerical simulation data are generated with Gaussian noise$N\left(0,0.1\right)$and resampled 100 times. Then, the hierarchical optimization for the other parameters $\alpha'_{12}$, $\alpha'_{21}$ and $\alpha'_{10}$ is verified and the results are summarized in **S13-15 Fig** and in **S4 Table** . As shown in **S13-15 Fig**, the fitting performance exceeds 0.98, demonstrating the feasibility of the optimization method. Additionally, the numerical results in Table 1 indicate that the optimization algorithm produces a small variance, highlighting its stability.


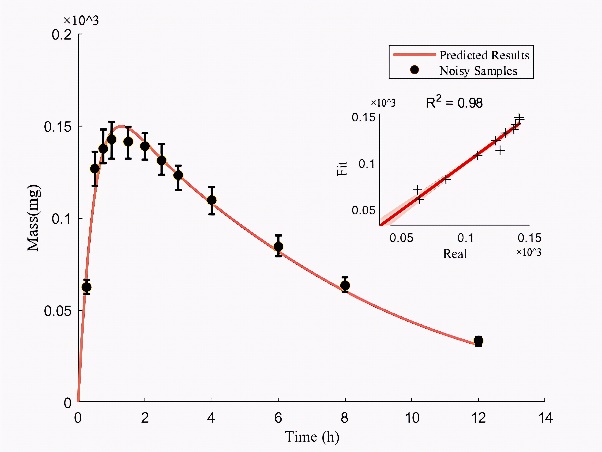


**S13 Fig. Noise samples and fitting curves in the central compartment over time-$\alpha'_{12}=1$.**


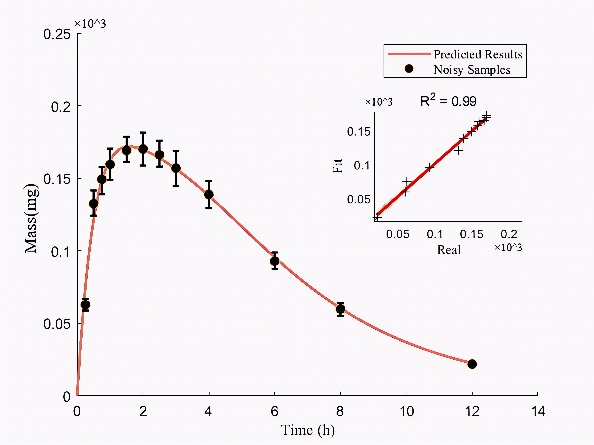


**S14 Fig. Noise samples and fitting curves in the central compartment over time-$\alpha'_{21}=1$.**


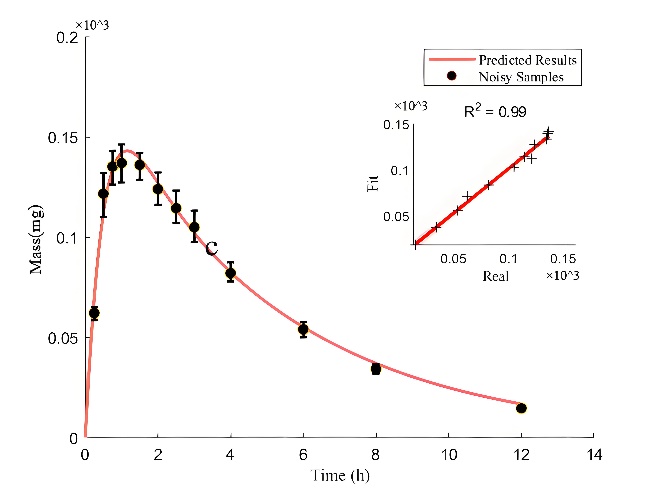


**S15 Fig. Noise samples and fitting curves in the central compartment over time-$\alpha'_{10}=1$.**

**S4 Table. Comparison of theoretical values and numerical solutions for drug X.**

| Interaction parameters (1/g•h)(95% CI) | | | | | | | | | | | | |
| --- | --- | --- | --- | --- | --- | --- | --- | --- | --- | --- | --- | --- |
| theoretical value | | | | numerical solution | | | | | | | | |
| $\alpha'_{00}$ | $\alpha'_{12}$ | $\alpha'_{21}$ | $\alpha'_{10}$ | $\alpha'_{00}$ | $\alpha'_{12}$ | | $\alpha'_{21}$ | | | | $\alpha'_{10}$ | |
| 0 | 1 | 0 | 0 | 0.0674  (0.0661,0.0687) | 0.9570 (0.9069,1.0071) | | -0.0182 (-0.0404,0.0040) | | | | 0.0452 (0.0402,0.0502) | |
| $\alpha'_{00}$ | $\alpha'_{12}$ | $\alpha'_{21}$ | $\alpha'_{10}$ | $\alpha'_{00}$ | $\alpha'_{12}$ | | $\alpha'_{21}$ | | $\alpha'_{10}$ | | | |
| 0 | 0 | 1 | 0 | 0.0115  (0.0101,0.0129) | 0.0801 (0.0439,0.1163) | | 1.0794 (1.0585,1.1003) | | -0.0436 (-0.0473,-0.0399) | | | |
| $\alpha'_{00}$ | $\alpha'_{12}$ | $\alpha'_{21}$ | $\alpha'_{10}$ | $\alpha'_{00}$ | | $\alpha'_{12}$ | | $\alpha'_{21}$ | | $\alpha'_{10}$ | |
| 0 | 0 | 0 | 1 | 0.0735 (0.0723,0.0747) | | 0.0277 (-0.0131,0.0685) | | 0.0064 (-0.0227,0.0355) | | 0.9623  (0.9532,0.9714) | |

**S5 Table . *Sensitivity* Analysis of the PSO Regularization Parameter** $\lambda$

| Interaction parameters()(95% CI) | | | | | | | | | | | |
| --- | --- | --- | --- | --- | --- | --- | --- | --- | --- | --- | --- |
| Drug | Interaction parameters | Theoretical value | PSO regularization parameter $\lambda$ | | | | | | | | |
|  |  |  | 0.1 | 0.2 | 0.3 | 0.4 | 0.5 | 0.6 | 0.7 | 0.8 | 0.9 |
| X | $\alpha_{00}^{'}$ | 0.4 | 0.331  (0.327, 0.335) | 0.355  (0.352, 0.358) | 0.377  (0.374, 0.380) | 0.455  (0.453, 0.457) | 0.465  (0.463, 0.467) | 0.4416  (0.4393,0.4439) | 0.463  (0.460,0.466) | 0.482  (0.479,0.485) | 0.501  (0.498, 0.504) |
|  | $\alpha_{12}^{'}$ | 0 | 0.0141  (0.0108,0.0174) | 0.0113  (0.0084,0.0142) | 0.0089  (0.0062,0.0116) | 0.0073 (0.0048,0.0098) | 0.0064  (0.0039, 0.0089) | 0.0059 (0.0033,0.0085) | 0.0067  (0.004,0.0093) | 0.0075  (0.0048, 0.0102) | 0.0088  (0.0059, 0.0117) |
|  | $\alpha_{21}^{'}$ | 0 | 0.0085  (0.0079,0.0091) | -0.0067  (-0.0073,-0.0061) | 0.0052  (0.0047,0.0057) | 0.0040  (0.0035,0.0045) | -0.0035  (-0.0039,-0.0031) | 0.0031 (0.0031,0.0031) | 0.0027  (0.002,0.0032) | -0.0024  (-0.0029,-0.0019) | 0.0020  (0.0014, 0.0026) |
|  | $\alpha_{10}^{'}$ | 0 | 0.0125  (0.0099,0.0151) | 0.0104  (0.0081, 0.0127) | 0.0087  (0.0066,0.0108) | 0.0079  (0.0060,0.0098) | 0.0075  (0.0057, 0.0093) | 0.0072 (0.0055,0.0089) | 0.0078  (0.006,0.0095) | 0.0086  (0.0068, 0.0104) | 0.0093  (0.0073, 0.0113) |

**S5 Table** presents the complete results corresponding to **Table 4** in the main text, showing the parameter estimates obtained by the PSO algorithm under different regularization coefficients (λ). Overall, as λ increases, parameter estimates become progressively more stable, and random fluctuations are markedly reduced. When λ is small (e.g., 0.1–0.3), several parameters deviate considerably from their theoretical values, indicating that the model is more susceptible to noise interference.

At λ=0.6, the estimated parameters are closest to the theoretical values and exhibit the smallest standard errors—for instance,$\alpha_{00}^{'}=0.4416\pm0.0043$—demonstrating stable fitting performance and good convergence. Further increases in λ still yield convergence but introduce slight bias, suggesting that excessive regularization may reduce model sensitivity.

Therefore, λ=0.6 can be regarded as the optimal setting, at which the model achieves an appropriate balance between estimation accuracy and robustness.

# 3 Model Application

As referenced in the main body of the paper [19-21], the experimental data are presented in **S6 and S7 Tables**.

**S6 Table. Experimental Data of Metoprolol and Captopril.**

| Time  (h) | Metoprolol  (single)  ng/ml) | Metoprolol  (combination)  (ng/ml) | Captopril  (single)  (ng/ml) | Captopril  (combination)  (ng/ml) |
| --- | --- | --- | --- | --- |
| 0.25 | 21.6$\pm$7.1 | 27.2$\pm9.8$ | 277.6$\pm$34.6 | 293.9$\pm$54.9 |
| 0.5 | 86.4$\pm$16.4 | 109.1$\pm$28.7 | 580.3$\pm$84.3 | 590.7$\pm$133.6 |
| 0.75 | 127.3$\pm$33.1 | 137.4$\pm$32.3 | 526.0$\pm$108.9 | 544.7$\pm$128.2 |
| 1 | 145.0$\pm$36.2 | 162.1$\pm$32.0 | 359.1$\pm$165.7 | 387.9$\pm$170.9 |
| 1.5 | 168.0$\pm$35.2 | 199.8$\pm$33.7 | 295.2$\pm$128.5 | 307.8$\pm$134.8 |
| 2 | 156.9$\pm$34.0 | 180.2$\pm$26.7 | 231.2$\pm$112.2 | 257.5$\pm$114.8 |
| 2.5 | 134.4$\pm$31.9 | 159.3$\pm$25.2 | 156.6$\pm$68.1 | 182.4$\pm$67.2 |
| 3 | 114.1$\pm$32.8 | 136.0$\pm$25.7 | 107.9$\pm$34.2 | 127.9$\pm$49.0 |
| 4 | 89.4$\pm$28.4 | 115.1$\pm$22.9 | 72.1$\pm$21.6 | 86.3$\pm$31.0 |
| 6 | 68.9$\pm$20.6 | 105.4$\pm$25.0 | 56.4$\pm$19.3 | 69.1$\pm$27.9 |
| 8 | 44.6$\pm$12.7 | 61.2$\pm$13.7 | 34.1$\pm$7.8 | 43.5$\pm$14.6 |
| 12 | 21.5$\pm$6.3 | 28.4$\pm$8.0 | 16.0$\pm$3.4 | 16.6$\pm$3.8 |

# Weight is 65 kg, the initial amount of Metoprolol is 50 mg, the initial amount of Captopril is 50 mg.

**S7 Table. Experimental Data of Imeglimin and Metformin.**

| Time  (h) | Imeglimin  (single) (ng/ml) | Imeglimin  (combination) (ng/ml) | Metformin  (single) (ng/ml) | Metformin  (combination) (ng/ml) |
| --- | --- | --- | --- | --- |
| τ | 0 | 793.4$\pm$48.5 | 276.2$\pm$25.2 | 355.8$\pm$32.6 |
| 0.5 | 759.8$\pm$67.5 | - | - | - |
| 1 | 1337.5$\pm$111.8 | 998.1$\pm$87.2 | 1284.8$\pm$95.1 | 1134.9$\pm$104.0 |
| 1.5 | 1564.4$\pm$137.0 | 1115.9$\pm$78.4 | 1311.4$\pm79.8$ | 1041.1$\pm$85.8 |
| 2 | 1712.2$\pm$129.1 | 1243.1$\pm$96.1 | 1270.1$\pm$77.3 | 944.1$\pm$72.0 |
| 3 | 1825.7$\pm$146.3 | 1351.6$\pm$97.8 | 1214.4$\pm$66.5 | 876.5$\pm$54.8 |
| 4 | 1658.1$\pm$117.6 | 1388.8$\pm$112.8 | 1170.3$\pm$53.2 | 803.0$\pm$56.4 |
| 5 | - | 1395.0$\pm$330.0 | 996.6$\pm$60.4 | 746.9$\pm$47.7 |
| 6 | 1021.2$\pm81.0$ | 1159.3$\pm$268.1 | 767.2$\pm$39.8 | 588.3$\pm$36.8 |
| 8 | 666.2$\pm$61.7 | 963.9$\pm$268.1 | 532.3$\pm$36.2 | 479.4$\pm$30.7 |
| 10 | 453.8$\pm$51.2 | 747.4$\pm$266.9 | 379.2$\pm$27.9 | 370.5$\pm$16.6 |
| 12 | 295.7$\pm$31.1 | 573.7$\pm$224.4 | 276.3$\pm$22.7 | 300.1$\pm$23.0 |

# Weight is 79 kg, the initial amount of Imeglimin is 1500 mg, the initial amount of Metformin is 850 mg.The symbol τ is used to represent a time point approaching zero but not identical to zero.

## 3.1 Model Results

The model rate parameters derived from the traditional PK model are listed in **S8 Table**, and the interaction parameters obtained from the coupled PK model are presented in **S9 Table**. Meanwhile, their fitting results are illustrated in **S16-18 Fig**.

**S8 Table. The rate parameters by traditional PK modeling.**

| Rate parameters ($h^{-1}$)(95% CI) | | | | |
| --- | --- | --- | --- | --- |
| Ref. | $\alpha_{00}$ | $\alpha_{12}$ | $\alpha_{21}$ | $\alpha_{10}$ |
| [19] | 0.205  (0.200, 0.210) | 84.192  (82.625, 85.759) | 8.122  (7.765, 8.479) | 15.162  (14.946, 15.378) |
| [20][21] | 0.268  (0.267, 0.269) | 231.984  (225.42, 238.54) | 4.519  (4.423, 4.615) | 28.344  (28.211, 28.477) |
| Ref. | $\beta_{00}$ | $\beta_{12}$ | $\beta_{21}$ | $\beta_{10}$ |
| [19] | 0.716  (0.700, 0.732) | 69.474  (68.334, 70.614) | 19.222  (18.407, 20.037) | 14.591  (14.458, 14.724) |
| [20][21] | 0.200  (0.195, 0.205) | 25.194  (22.972, 27.416) | 1.299  (1.038, 1.560) | 18.969  (18.777, 19.161) |
| Ref. | $Vc$(ml) | | | |
| [19] | 4163.137 (4147.268, 4179.006) | | | |
| [20][21] | 4721.361 (4703.999, 4738.723) | | | |

**S9 Table. The interaction parameters of coupled PK modeling.**

| Interaction parameters()(95% CI) | | | | |
| --- | --- | --- | --- | --- |
| Ref. | $\alpha'_{00}$ | $\alpha'_{12}$ | $\alpha'_{21}$ | $\alpha'_{10}$ |
| [19] | -1.270  (-1.387, -1.153) | -729.2  (-784.5, -673.9) | 585.2  (475.1, 695.3) | -4854  (-5037.1, -4670.9) |
| [20][21] | -0.178  (-0.180, -0.176) | 9945  (9618.4, 10271.6) | -2.729  (-3.219, -2.239) | -45.004  (-91.647, 1.639) |
| Ref. | $\beta'_{00}$ | $\beta'_{12}$ | $\beta'_{21}$ | $\beta'_{10}$ |
| [19] | -2.354  (-2.455, -2.253) | -9704  (-10230.4, -9177.6) | 4905  (4725.2, 5084.8) | 2481  (2351.0, 2611.0) |
| [20][21] | -0.062  (-0.073, -0.051) | -3.224  (-4.359, -2.089) | -4.141  (-4.205, -4.077) | 1.13  (1.119, 1.141) |


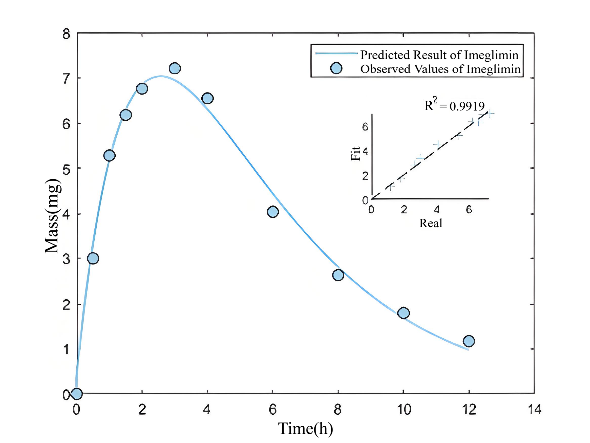


**S16 Fig.The fitted curve of drug** amount **in the central compartment over time- Imeglimin alone.**


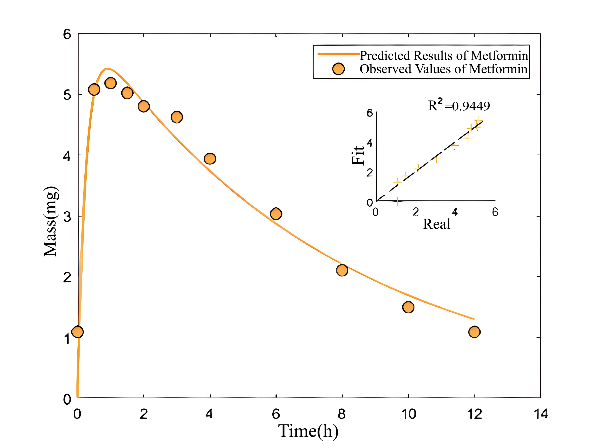


**S17 Fig.The fitted curve of drug** amount **in the central compartment over time- Metformin alone.**


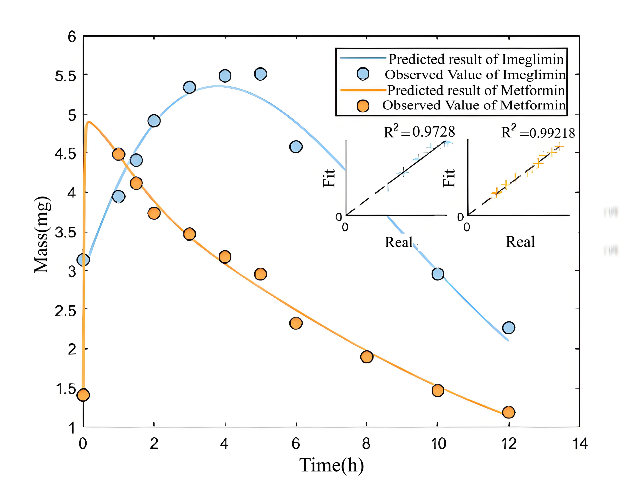


**S18 Fig.The fitted curve of drug** amount **in the central compartment over time- oral co-administration.**

**S16-18 Fig** shows that the fitting performance$R^{2}$is 0.9728 for Imeglimin and 0.99218 for Metformin. Moreover, the subsequent section analyzes the coupling parameters and their interaction effects on both drugs. This analysis aims to provide a deeper understanding of the pharmacokinetic interactions between Imeglimin and Metformin, as well as their implications for therapeutic applications.

## 3.2 Parameter Analysis

Metformin facilitates the transfer of Imeglimin from the central compartment to the peripheral compartment while inhibiting its transfer from the peripheral compartment back to the central compartment, thereby increasing the proportion of Imeglimin in the peripheral compartment. Concurrently, Metformin inhibits the absorption and elimination of Imeglimin, prolonging its duration of action within the body.

Conversely, Imeglimin inhibits the absorption of Metformin and promotes its elimination, thereby reducing the residence time of Metformin in the body. This reduction in duration may potentially mitigate Metformin-associated side effects, such as lactic acidosis.

**(1) The effect of Metformin on** **Imeglimin**

Parameter $\alpha'_{00}$, characterized by a negative value, indicates that Metformin acts as an inhibitor of Imeglimin absorption. The highest inhibition observed for parameter $\alpha'_{00}$ is 56.46%. The average inhibition of Imeglimin transfer from the central to the peripheral compartment at 2 h, 6 h, and 12 h is 50.48%, 39.57%, and 27.44%, respectively.

Parameter $\alpha'_{12}$, which has a positive value, demonstrates that Metformin facilitates the transfer of Imeglimin from the central to the peripheral compartment. The maximum facilitation effect for parameter $\alpha'_{12}$ is 19.22%. The average facilitation rates at 2 h, 6 h, and 12 h are 18.91%, 15.21%, and 11.38%, respectively.

The negative value of parameter$\alpha'_{10}$ suggests that Metformin inhibits the elimination of Imeglimin. The maximum suppression effect recorded is 0.71%, and the mean inhibitory effects at 2 h, 6 h, and 12 h are 0.70%, 0.56%, and 0.42%, respectively.

Due to the lack of amount data for Metformin in the peripheral compartment, it is not feasible to determine the maximum inhibitory effect from the experimental data. However, using the model presented in this study to simulate the amount of both drugs in the peripheral compartment, the average inhibition of Imeglimin transfer from the peripheral compartment to the central compartment by Metformin at 2 h, 6 h, and 12 h was found to be 1.88%, 1.07%, and 0.87%, respectively.

**(2) The effect of Imeglimin on Metformin**

The negative value of parameter $\beta'_{00}$ illustrates that Imeglimin reduces Metformin absorption. The greatest inhibitory effect for parameter $\beta'_{00}$ is 46.50%, while the average inhibition values at 2 h, 6 h, and 12 h are 41.08%, 31.09%, and 20.48%, respectively.

For parameter $\beta'_{12}$, a negative value shows that Imeglimin impedes the transfer of Metformin from the central compartment to the peripheral compartment. The highest inhibition for parameter $\beta'_{12}$ is observed at 0.071%, with average inhibition at 2 h, 6 h, and 12 h being 0.052%, 0.048%, and 0.042%, respectively.

Parameter $\beta'_{10}$, though negative, reveals that Imeglimin facilitates Metformin elimination. The maximum facilitation for parameter $\beta'_{10}$ is 0.033%. The average facilitation values at 2 h, 6 h, and 12 h are 0.024%, 0.029%, and 0.025%, respectively.

Since amount data for Imeglimin in the peripheral compartment were not measured, estimating the maximum inhibitory effect based solely on experimental data is also not possible. Nevertheless, simulations using the model indicate that the average inhibition of Metformin transfer from the peripheral compartment to the central compartment by Imeglimin at 2 h, 6 h, and 12 h was 76.06%, 90.09%, and 77.06%, respectively.
