# Supplementary material for: Coupled pharmacokinetic model unveils drug-drug interactions in plasma concentration
Source: PLoS One. 2026 Feb 10;21(2):e0339052. doi: 10.1371/journal.pone.0339052 (PMC12890151; doi:10.1371/journal.pone.0339052)
Supplement: Attachment — Submitted filename: Response to Reviewers.docx [file pone.0339052.s004.docx]

**Response to Reviewers' Comments**

**Dear Editor and Reviewers,**

Thank you for your continued consideration of our manuscript ”Coupled Pharmacokinetic Model Unveils Drug-Drug Interactions in Plasma Concentration” and for your helpful suggestions during the revision process. We have carefully addressed all the comments and revised the manuscript accordingly, and have further supplemented and optimized the relevant analysis during this period.

We sincerely inform the editorial department of a change in authorship. The research content of this thesis involves a lot of computer program design and simulation modeling work, and the original first author is mainly responsible for the development and simulation analysis of related programs. However, due to personal reasons, the original first author has left the research group and cannot continue to participate in the subsequent paper revision and improvement work.

To ensure the continuity of the manuscript and the completeness of the research results, we have invited another number of the research team- Hong Huang - to join the author team and serve as the first author, with full knowledge from the original author and unanimous consent from all authors. The author has undertaken core tasks such as data review, algorithm validation, model simulation, and paper revision in subsequent work.

This author adjustment is entirely based on the practical needs of scientific research work and does not involve any authorship disputes or academic disputes. All authors have signed a consent form to confirm the author change and the final author order. The signed document has been submitted along with the letter.

Please kindly understand the objective reasons for this adjustment and continue to review our revised manuscript. We would like to express our gratitude once again to the editors and teachers of your journal for their understanding, support, and hard work!

Finally, we have provided a point-by-point response to all comments. In the submitted "Revised manuscript with Track Changes" version, additions and changes are indicated in red font, and all line and page numbers refer to the revised manuscript.

Best wishes.

Corresponding author: Weifeng Jin; Xiaohong Li

E-mail : [jin_weifeng@126.com](mailto:jin_weifeng@126.com) (WFJ);[li_xiaoh2005@163.com](mailto:li_xiaoh2005@163.com) (XHL)

**Response to Comments from the editor:**

**Comment 1:Innovation and Background**

The coupled PK model introduces interaction terms and parameter heterogeneity, but the author should more clearly compare it with PBPK-DDI and nonlinear mixed effects models, which are already able to explain variability and interactions. Statements like 'existing PK models assume constant parameters' are too simplistic and ignore existing nonlinear, time-varying, and interactive models. Unless explained through updated references, it will weaken the innovation claim.

**Response:** Traditional compartmental models typically assume that pharmacokinetic parameters, such as clearance rate and distribution volume, remain constant over time and drug concentration to simplify the model structure and facilitate parameter estimation. However, this assumption is difficult to capture the dynamic dependence of parameters over time or concentration. In recent years, physiology based pharmacokinetic models (PBPK) have been able to describe such temporal variability and interactivity to some extent, but they often rely heavily on prior information and complex computational frameworks. The coupled PK model proposed in this study introduces interaction terms into the concise structure of the classical compartmental model to capture the dynamic coupling effects of drug concentration dependence, thereby achieving a balance between model complexity and explanatory power, providing an intermediate solution between traditional compartmental models and PBPK models for drug interaction research.

It should be pointed out that the establishment of any model is based on certain approximations and assumptions. Simplifying the system to a linear process or assuming constant parameters within a specific dose range is a reasonable approximation that facilitates theoretical derivation and parameter identification. However, when the research scope expands to a wider concentration range or non-linear processes dominate, more complex non-linear or time-varying models need to be adopted to improve the accuracy of characterization.

**Changes in manuscript :** Row number 69-76:

PK modeling of drug combinations typically employs compartmental models, where traditional compartmental models often assume that pharmacokinetic parameters (e.g., clearance, volume of distribution) remain constant over time and drug concentration to simplify model structure and facilitate parameter estimation. [12-14] However, this assumption struggles to capture the dynamic dependencies arising from parameter changes over time or concentration. In recent years, physiologically based pharmacokinetic (PBPK) models have demonstrated some capability to describe such time-varying and interactive properties, yet they often rely on extensive prior information and complex computational frameworks[15].

**Comment 2. Biological rationality of the model**

The model assumes a linear relationship between the quality of one drug and the PK parameters of another drug. This is questionable because many DDIs involve nonlinear processes such as enzyme saturation and transporter inhibition. Some parameter estimates are extremely large or negative (such as -729.2, -9704, 4905, -4854 in Table 5), which raises questions about whether these values are physiologically interpretable or merely mathematical artifacts. The author should clarify the clinical significance of these coefficients and indicate whether constraints should be added to ensure their validity.

**Response:** (1)Why is the model linear: The linear relationship between the PK parameters of a drug and another drug is mainly based on the model hypothesis of this study. Similar to treating extremely small segments of a curve as straight lines when calculating curve integrals, drug interactions within a specific dose range can be understood as linear relationships. Of course, when the research scope expands to a wider concentration range or nonlinear processes dominate, the accuracy of nonlinear models is indeed higher.

**Changes in manuscript:** Row number:93-101

In this study, the absorption, distribution, and elimination rates of one drug are considered to be influenced by monotonic changes in the mass of the other drug. In addition, this study is based on an important assumption that it is only applicable to specific dose ranges. Similar to treating extremely small segments of a curve as straight lines when calculating curve integrals, the heterogeneity of pharmacokinetic model parameters is defined as a linear relationship, with the sign of the slope indicating their interaction. For clarity, X will be used to represent one drug and Y to represent the other. The compartmental structure of the coupled PK model is illustrated in **Fig 1**. Based on the assumptions of parameter heterogeneity and linear relationship, the two-compartment coupled PK model is presented as follows,the specific form is shown in **Eq 1**.

**Response:** (2)Why are parameters negative: In the model of this study, negative parameters are used as antagonistic coefficients to quantify the inhibitory effect of one drug on the absorption rate or degree of another drug. On the contrary, positive numbers have a promoting effect.

**Changes in manuscript:** Row number:146-153

In coupled PK models, some common strategies include enhancing the absorption of drug X in the central compartment and promoting its transfer from the peripheral compartment to increase the mass of drug X in the central compartment. Inhibiting the elimination of drug X and reducing its transfer from the central compartment to the peripheral compartment are also effective strategies. According to the structure of the model, the interaction parameters can be either positive or negative. The negative parameter serves as the inhibitory coefficient, quantifying the inhibitory effect of one drug on the absorption rate or degree of another drug. On the contrary, the positive parameter is the promotion coefficient.

**Response:** (3)Why are the parameters extremely large: The parameters are mainly concentrated in the original Table 2 and the original Table 5, where Table 2 shows the various levels of parameters under single drug action, and Table 5 shows the interaction parameters under the combined action of two drugs. There are two reasons for the large parameters. One is that the time unit selected for this study is per hour (h), and the amount of medication transported from one compartment to another per hour is a cumulative process, with larger values being a normal phenomenon; The second reason is that the mass unit selected in this study is grams (g), but in reality, the dosage of the two drugs is often measured in milligrams (mg) due to the inconsistency between their defined unit (g) and the input data unit (mg). To compensate for the 10^3^ fold difference in quality units, the parameter estimates are proportionally enlarged, which is the result of mathematical processing and does not affect the biological interpretation of the model itself.

**Comment 3. Robustness and validation of optimization methods**

The method of layering+particle swarm optimization (PSO) has novelty, but lacks benchmark comparison. The author should compare parameter recovery and stability with standard nonlinear regression or mixed effects methods such as NONMEM and Monolix. It is currently unclear whether PSO and regularization improve robustness in noisy data, or whether simpler maximum likelihood/Bayesian methods can achieve similar results. Sensitivity analysis is needed for λ (regularized hyperparameter) - is the fitting result stable under different values?

**Response: (1)The necessity and rationality of hierarchical modeling**

This study adopts a hierarchical structure mainly based on considerations of model complexity and parameter identification. If all drug parameters are directly modeled together, it will involve 16 parameters to be estimated, with a high dimensionality, which can easily lead to convergence difficulties and excessive computational burden. Therefore, this study divides the parameter system into two layers: the first layer is the basic parameters for single drug administration, and the second layer is the interaction parameters for the combination of two drugs. The parameters of a single drug can be directly obtained through analysis, without relying on optimization processes, thereby significantly reducing the subsequent optimization dimensions while maintaining physiological interpretability. This hierarchical strategy not only improves the efficiency and stability of model solving, but also helps to more clearly reveal the hierarchical relationship between single drug and combination effects, enhancing the interpretability of the model.

**Changes in manuscript:** Row number:232-244

In order to effectively apply the model, it is crucial to accurately estimate the model parameters from experimental data. If all drug parameters are directly modeled together, it will involve 16 parameters to be estimated, with non-linear complexity and a large number of parameters. Traditional parameter determination methods may be insufficient. For this purpose, the parameter system is divided into two layers: the first layer consists of pharmacokinetic parameters related to a single drug [18], independently estimated from experimental data, and the second layer consists of interaction parameters for combination therapy. The workflow of these steps is shown in **Fig 3**. The parameters of a single drug can be directly obtained through experimental data analysis, without relying on optimization processes, thereby significantly reducing the subsequent optimization dimensions while maintaining physiological interpretability. This hierarchical strategy not only improves the efficiency and stability of model solving, but also helps to more clearly reveal the hierarchical relationship between single drug and combination effects, enhancing the interpretability of the model.

**Response: (2)The basis for selecting Particle Swarm Optimization (PSO) algorithm**

In the modeling context of this study (nonlinear differential equation model, lack of analytical solutions, parameter estimation through experimental data), particle swarm optimization (PSO) method is more applicable than maximum likelihood estimation (MLE) and Bayesian inference. The main reason is that such models are usually non-convex, non-differentiable, or numerically unstable. Traditional MLE relies on a continuously differentiable likelihood function, which is susceptible to the influence of initial values and local extremum; Although Bayesian methods can provide parameter uncertainty assessment, they require repeated numerical integration in high-dimensional parameter spaces, resulting in significant computational overhead. In contrast, PSO, as a global optimization algorithm based on swarm intelligence, does not require gradient information and can directly optimize parameters based on model numerical solutions. It has strong robustness and global convergence ability, making it more efficient and robust in parameter estimation of complex nonlinear dynamic models.

**Changes in manuscript:** Row number:347-358

In the modeling context of this study (nonlinear differential equation model, lack of analytical solutions, parameter estimation through experimental data), particle swarm optimization (PSO) method is more applicable than maximum likelihood estimation (MLE) and Bayesian inference. The main reason is that such models are usually non-convex, non-differentiable, or numerically unstable. Traditional MLE relies on a continuously differentiable likelihood function, which is susceptible to the influence of initial values and local extremum; Although Bayesian methods can provide parameter uncertainty assessment, they require repeated numerical integration in high-dimensional parameter spaces, resulting in significant computational overhead. In contrast, PSO, as a global optimization algorithm based on swarm intelligence, does not require gradient information and can directly optimize parameters based on model numerical solutions. It has strong robustness and global convergence ability, making it more efficient and robust in parameter estimation of complex nonlinear dynamic models.

**Response: (3) Analysis of Parameter Stability and Regularization Sensitivity**

This study has conducted systematic verification on the sensitivity of the regularization hyperparameter λ mentioned in the review comments. We gradually tested within the range of λ=0.1 to 0.9, and the results showed that the model fitting effect was the best and the results were the most stable at λ=0.6, with the least fluctuation in parameter estimation. This result indicates that the regularization term can balance the smoothness and robustness to noise of the model under moderate intensity. In addition, the stable convergence of PSO under multiple sets of λ values further proves the repeatability and robustness of its optimization process. Therefore, it can be considered that this method maintains good parameter stability and identification reliability under different regularization strengths.

The revised manuscript has added a section 3.3 on model performance comparison, and the new Table 3 can demonstrate the stability of PSO regularization parameters in the future.

**Changes in manuscript:** Row number:343

**Table 4. Sensitivity Analysis of the PSO Regularization Parameter** λ

| Interaction parameters (1·10^3^/(mg·h)) | | | | | | | |
| --- | --- | --- | --- | --- | --- | --- | --- |
| Drug | Interaction parameters | Theoretical value | | PSO regularization parameter λ | | | |
|  |  |  |  | 0.1 | 0.3 | 0.6 | 0.9 |
| *X* | $\alpha_{00}^{'}$ | | 0.4 | 0.331 ±0.007 | 0.377 ±0.005 | 0.4416 ±0.0043 | 0.501 ±0.006 |
|  | $\alpha_{12}^{'}$ | | 0 | 0.0141 ±0.0062 | 0.0089 ±0.0050 | 0.0059 ±0.0048 | 0.0088 ±0.0055 |
|  | $\alpha_{21}^{'}$ | | 0 | 0.0085 ±0.0012 | 0.0052 ±0.0010 | 0.0031 ±0.0000 | 0.0020 ±0.0011 |
|  | $\alpha_{10}^{'}$ | | 0 | 0.0125 ±0.0048 | 0.0087 ±0.004 | 0.0072 ±0.0031 | 0.0093 ±0.0038 |

**Comment 4. Use of real data**

The selected dataset ([19-21]) has a small sample size and heterogeneity (n=12, n=16). This raises doubts about the accuracy of parameter estimation. The method of converting plasma concentration into "drug quality" is not common, and more explanation is needed for the rationality of this conversion (formula 2). Some datasets lack peripheral ventricular measurements, but the model estimates peripheral related parameters. The assumptions regarding these missing data should be clearly stated.

**Response:** (1)**Small sample size and heterogeneity**: The selected dataset for this study has a small sample size and heterogeneity, which may affect the accuracy of model parameter estimation. We recognize this and have explicitly stated in the revised paper that the current research results are only preliminary explorations and will need to be validated through larger sample sizes in the future. In addition, we will also consider the heterogeneity of the samples in the analysis and control for confounding factors through appropriate statistical methods such as stratified analysis, mixed effects models, etc., in order to reduce the impact of heterogeneity on the results.

**Changes in manuscript:** Row number:602-606

The current selected dataset has a small sample size and high heterogeneity. Although potential confounding factors have been controlled through appropriate statistical methods such as stratified analysis and mixed effects models to reduce the impact of heterogeneity on the results, the results of this study are still only preliminary explorations. Future research should be validated through larger sample sizes of data to further confirm the effectiveness and universality of the model.

**Response:** (2)**The rationality of the conversion formula between plasma concentration and drug mass**: The rationality of this conversion can be explained from the following two aspects: first, the law of conservation of mass, which states that the mass of a drug is always equal to the product of concentration and volume; The second is the perspective of parameter definition. In pharmacokinetics, the apparent distribution volume Vc does not refer to an actual physical volume, but describes the distribution characteristics of drugs in the body. Specifically, Vc represents that if the drug is evenly distributed within this volume, the drug concentration in the plasma will be equal to the actual measured concentration cx. Therefore, Vc is a hypothetical theoretical quantity that helps us better understand the distribution of drugs in the body.

**Changes in manuscript:** Row number:136-144

The rationality of this formula conversion comes from two aspects. Firstly, the law of conservation of mass states that the mass of a drug is always equal to the product of concentration and volume; The second is the perspective of parameter definition. In pharmacokinetics, the apparent distribution volume *Vc* does not refer to an actual physical volume, but describes the distribution characteristics of drugs in the body. Specifically, *Vc* represents that if the drug is evenly distributed within this volume, the drug concentration in the plasma will be equal to the actual measured concentration $c_{x}$. Since the parameters of the experimental subjects can be assumed to be equal in both single pharmacokinetic and coupled pharmacokinetic programs, the experimental data of the single pharmacokinetic program can be analytically solved [17].

**Response: (3)Peripheral chamber related parameters**: In this study, due to the lack of direct measurement data of the peripheral chamber, we referred to the typical range of apparent distribution volume in relevant literature and set reasonable parameter boundary conditions to ensure the stability of the model and the rationality of the results. In addition, during the model fitting process, we applied reasonable boundary constraints on peripheral ventricular parameters based on the distribution characteristics of drugs to avoid unrealistic results, and clearly pointed out the limitations of these assumptions in the discussion section. Although these assumptions may have an impact on the final results, they are based on existing literature and theoretical support, and can provide valuable preliminary estimates for understanding the distribution of drugs in the body.

**Changes in manuscript:** Row number:281-286

In addition, regarding the direct measurement data of the peripheral chamber, the typical distribution range of these parameters in relevant literature was also referred to, and reasonable parameter boundary conditions were set in the fitting to ensure the stability of the model and the rationality of the results. These parameters are based on existing literature and theoretical support, and can provide valuable preliminary estimates for understanding the distribution of drugs in the body.

**Comment 5: Explanation and clinical relevance**

The model output is transformed into mechanism explanations (such as "Captopril inhibits metoprolol absorption/elimination"), but considering the irrationality of parameter values and data limitations, these statements may be overinterpreted. The clinical significance (such as dose adjustment, DDI risk assessment) is only briefly mentioned. If we can clearly discuss how the model supports regulatory or treatment decisions, rather than just academic modeling, it will enhance the value of the paper.

**Response**: (1)**The caution in explaining the model mechanism**: The parameter "irrationality" mentioned in the article is actually a mathematical representation based on dimensional processing, rather than the model itself having irrationality. In terms of data limitations, although there is indeed a problem of small sample size in this article, the initial nature of the mechanism discussion has been clearly stated in the original manuscript, and the conclusions of the model are supported by existing literature.

**Changes in manuscript**: Row number:554-562

The dataset used in this study has a relatively limited sample size and exhibits certain individual heterogeneity. Although a hierarchical estimation and mixed effects framework was used to control for potential confounding factors, it should be acknowledged that the conclusions obtained are only exploratory. The setting of peripheral chamber related parameters is mainly based on the empirical range of apparent distribution volume in literature, and reasonable boundary conditions are set to ensure model stability. Although this method has theoretical and empirical support, due to the lack of direct measurement data, there may still be biases in the relevant estimates, and there is an urgent need for larger sample sizes and multi center datasets to validate and improve the model's universality and extrapolation.

**Response:** (2)**The clinical significance and improvement of the model**: The revised manuscript carefully revised some mechanistic statements, avoided overly speculative components, and removed inferences lacking direct experimental or literature basis. At the same time, the discussion section emphasized the potential clinical and regulatory value of the coupled PK model, especially in the risk assessment of drug combination use, which has important regulatory significance. This method can serve as a powerful supplementary tool for PBPK or population PK models, especially suitable for situations where sample sizes are insufficient or data is scarce. Through this model, preliminary quantitative support can be provided for risk assessment of drug combination therapy, thereby providing valuable references for regulatory agencies and clinical decision-making. By further validating the applicability of the model in different drug combination scenarios, new tools and methods can be provided for dose adjustment and drug interaction (DDI) risk assessment in clinical treatment in the future.

**Changes in manuscript:** Row number:578-583

Clinical and regulatory significance: The coupled PK model can achieve quantitative evaluation of potential drug interactions in the early stages of drug development, providing basic support for clinical dose optimization and adverse reaction prediction. This framework can also provide auxiliary quantitative tools for drug regulatory agencies to conduct drug interaction risk assessment and label revision in the review process, thereby enhancing the practical application value of the model at the scientific decision-making level.

**Response to Comments from Reviewer # 1:**

**Comment 1. Symbol clarity:** Currently, the mathematical expressions of formulas (1) and (3) in the PDF are difficult to read, and there are symbols that are not clear

**Response:** The symbols have now been corrected and are very clear.

**Comment 2. PBPK extension**: The author limited the study to the atrioventricular model, but mentioned possible extensions of PBPK modeling. If a brief overview of the challenges of PBPK and the elements to be included could be provided, it would enhance the discussion;

**Response:** We have added a discussion of the potential challenges of PBPK modeling.

**Changes in manuscript:** Row number:567-577

Model system expansion: Based on the atrioventricular model framework, it is further developed into a physiological pharmacokinetic (PBPK) model to achieve more physiologically relevant pharmacokinetic descriptions. The main challenges of PBPK model include: (1) large parameter dimensions and complex transport processes between different organizations; (2) There are significant differences in individual physiological structure, hemodynamics, metabolic enzymes, and transporter activity; (3) The source of parameters relies on literature inference or experimental extrapolation, which has significant uncertainty. Future models can gradually incorporate key physiological and biochemical factors, such as tissue blood flow distribution coefficient, enzymatic reaction parameters, transmembrane transport rate, plasma protein binding rate, liver and kidney clearance pathways, and disease state correction factors, to improve the physiological authenticity and predictability of the model.

**Comment 3**. Sensitivity of interaction parameters: Some terms (such as α₁₂′, β₁₂′) are close to zero and have large differences. If these items are included in the system

The calculation is not significant, its significance should be discussed, and deletion should be considered to increase the simplicity of the model.

**Response:** The setting of $\alpha_{00}^{'}=0.4$ in old Table 2 and other interaction parameters being zero does not mean that only $\alpha_{00}^{'}$ has biological significance, but rather adopts an experimental design strategy of "controlling variables (fixed parameters)". This processing aims to validate the principle of the model by fixing other parameters at zero level and observing the effect of $\alpha_{00}^{'}$ changes on pharmacokinetic behavior in isolation. When the simulation results show that the change in this parameter can trigger a concentration time trend that conforms to pharmacological common sense (such as an earlier peak time and an increase in peak concentration of drug X in the central chamber), the rationality and explanatory power of the model structure setting are mathematically verified. Similar validations were conducted on other interaction parameters in the study, and their trend charts are included in the appendix.

**Response to Comments from Reviewer # 2:**

**Comment 1. Accuracy of pharmacological statements**

Some of the statements regarding DDI are inconsistent with the literature. For example, imeglimin has not been shown to prolong the half-life of metformin or significantly reduce its concentration in clinical practice. Similarly, the study on the interaction between cimetidine and imigliptin concluded that there was no significant effect. Metoprolol relies on CYP2D6 metabolism, but Captopril is mainly excreted through the kidneys and not metabolized by CYP2D6.

Operation: Correct these descriptions to ensure that all pharmacological statements are supported by current references; If the evidence is limited, please maintain a neutral explanation.

**Response:** The pharmacological statements without reference support have been removed from the revised manuscript, and a neutral explanation has been adopted in the derivation of the mechanism. The specific content can be found in 4.3 Parameter Analysis.

**Changes in manuscript:** Row number:507-524

Cap and Met seem to mutually inhibit each other's transport from the central chamber to the peripheral chamber, while tending to promote processes in opposite directions. This pattern may partially explain the phenomenon of increased distribution of drugs in the central compartment [22]. This result is somewhat consistent with the pharmacological effects of both drugs [23-24] - they both act on vascular receptors, causing vasodilation and lowering blood pressure. Therefore, the simulation results provide a possible mechanism explanation for the pharmacokinetic process, consistent with the known pharmacological characteristics of the two drugs.

Cap may mainly act by affecting the metabolism and excretion of Met, as suggested in reference [19]. Met is mainly metabolized in the liver, with only a small amount excreted in its original form through urine. Although Cap is not a typical liver enzyme inhibitor, both are metabolized by cytochrome P450 2D6 [25-27]. Therefore, simultaneous administration may interfere with the metabolism of Met, leading to a decrease in its clearance rate and a slight increase in plasma concentration.

On the contrary, the model results show that Met has almost no significant effect on the elimination of Cap. Due to the fact that Cap is metabolized in the liver and mainly excreted through the kidneys, slight liver function regulation usually does not cause significant changes in its pharmacokinetic characteristics. Therefore, when combined with Met, the pharmacokinetic characteristics of Cap remained generally stable.

**Comment 2. Model structure, scale, and units**

The partially estimated rate constants and interaction terms appear unreasonable compared to the standard PK parameters. It is unclear whether the central chamber volume (Vc) is drug specific or shared. The unit has not always been clear.

Operation: Provide unit checks for all parameters, demonstrate the rationality of parameter sizes using literature scope, and clarify the handling of Vc. The demonstration model can generate realistic predictions at clinical doses (mg).

**Response:** (1)The reason why the parameters in this study are unreasonable compared to the standard PK parameters is due to unit issues. One is that the time measurement unit is set to per hour (h), which is an accumulated process that can cause the parameters to become too large; The second is to set the unit of mass measurement as grams (g) instead of milligrams (mg), which will result in a 10^3^ fold scaling of the parameter.

**Response:** (2) The central chamber volume Vc is shared. Its valuation processing is as follows: Vc in this article is estimated by fitting from plasma concentration time data of single drug administration using stratified optimization techniques. Specifically, in the first step of modeling, the experimentally measured blood drug concentration is connected to the central compartment drug mass predicted by the model using the relationship $C(t)=Ac(t)/Vc$. Then, the particle swarm optimization algorithm is used to simultaneously optimize the parameter set including Vc and various rate constants in the traditional compartment model framework, in order to minimize the error between the model prediction curve and the experimental data; The optimal Vc estimate obtained from this was used as a known fixed parameter in the subsequent analysis of the interaction between combined administration to ensure the stability of model identification.

**Response:** (3)The revised manuscript has changed all drug dosage units in the article to milligrams.

**Comment 3. Recognizability and parameter estimation**

The model introduces many interaction terms, which raises concerns about excessive parameterization. There is no evidence to suggest that the parameters are identifiable under a given amount of data.

Operation: Conduct and report recognizability analysis (such as likelihood profile, bootstrap, Bayesian posterior distribution). Provide confidence intervals and correlations for parameter estimation. If the recognizability is poor, the model structure should be simplified (for example, limited to the interaction between absorption and clearance).

**Response:** The revised manuscript has added a section on model performance comparison. Table 4 uses bootstrap and samples 100 time points. The null hypothesis is that there is no statistical difference between the numerical solution and the theoretical solution, and the final P-values are all greater than 0.05, indicating the stability and reliability of the results.

**Comment 4. Statistical analysis and validation**

The selection of λ is subjective, and the evaluation is almost entirely dependent on R ². More rigorous statistical methods are needed.

Operation: Replace heuristic lambda selection with cross validation or AIC/BIC. Add diagnostic maps (weighted residuals, prediction checks, prediction error indicators). Report uncertainty for all fitting parameters of synthetic data and clinical data.

**Response:** The revised manuscript has added a section on model performance comparison, and Table 4 has conducted sensitivity analysis on λ. The results show that when λ=0.6, the model accuracy is higher and more stable.

**Changes in manuscript:** Row number:343、345

**Table 4. Sensitivity Analysis of the PSO Regularization Parameter** λ

| Interaction parameters (1·10^3^/(mg·h)) | | | | | | | |
| --- | --- | --- | --- | --- | --- | --- | --- |
| Drug | Interaction parameters | Theoretical value | | PSO regularization parameter λ | | | |
|  |  |  |  | 0.1 | 0.3 | 0.6 | 0.9 |
| *X* | $\alpha_{00}^{'}$ | | 0.4 | 0.331 ±0.007 | 0.377 ±0.005 | 0.4416 ±0.0043 | 0.501 ±0.006 |
|  | $\alpha_{12}^{'}$ | | 0 | 0.0141 ±0.0062 | 0.0089 ±0.0050 | 0.0059 ±0.0048 | 0.0088 ±0.0055 |
|  | $\alpha_{21}^{'}$ | | 0 | 0.0085 ±0.0012 | 0.0052 ±0.0010 | 0.0031 ±0.0000 | 0.0020 ±0.0011 |
|  | $\alpha_{10}^{'}$ | | 0 | 0.0125 ±0.0048 | 0.0087 ±0.004 | 0.0072 ±0.0031 | 0.0093 ±0.0038 |

**Table 5. Results under bootstrap**

| Interaction parameters (1·10^3^/(mg·h)) | | | | |
| --- | --- | --- | --- | --- |
| Drug | Interaction  parameters | Theoretical  value | Numerical solution  (95% *CI*) | *P*  *Value* |
| *X* | $\alpha_{00}^{'}$ | 0.4 | 0.40  (0.37,0.43) | 0.634 |
|  | $\alpha_{12}^{'}$ | 0 | 0.01  (-0.02,0.04) | 0.056 |
|  | $\alpha_{21}^{'}$ | 0 | 0.00  (-0.03, 0.03) | 0.300 |
|  | $\alpha_{10}^{'}$ | 0 | 0.00  (-0.03, 0.03) | 0.268 |

**Comment 5. Handling of unobserved rooms and chambers**

The practice of replacing unknown peripheral mass with the maximum observed value of other chambers is a temporary method and may introduce bias.

Operation: Provide stronger theoretical explanations or redesign constraints to avoid overestimating interaction effects.

**Response:** In this study, due to the lack of direct measurement data of the peripheral chamber, we referred to the typical range of apparent distribution volume in relevant literature and set reasonable parameter boundary conditions to ensure the stability of the model and the rationality of the results. In addition, during the model fitting process, we applied reasonable boundary constraints on peripheral ventricular parameters based on the distribution characteristics of drugs to avoid unrealistic results, and clearly pointed out the limitations of these assumptions in the discussion section. Although these assumptions may have an impact on the final results, they are based on existing literature and theoretical support, and can provide valuable preliminary estimates for understanding the distribution of drugs in the body.

**Changes in manuscript:** Row number:281-286

In addition, regarding the direct measurement data of the peripheral chamber, the typical distribution range of these parameters in relevant literature was also referred to, and reasonable parameter boundary conditions were set in the fitting to ensure the stability of the model and the rationality of the results. These parameters are based on existing literature and theoretical support, and can provide valuable preliminary estimates for understanding the distribution of drugs in the body.

**Comment 6. Data availability and transparency**

The manuscript relies on published data, but does not provide a digital dataset or analysis code. The current data availability statement does not meet the requirements of the journal.

Operation: Store all digitized plasma concentration time curves, extraction scripts, and analysis codes in a public database. Update the data availability statement and attach the link.

**Response:** The data is shown in the attachment, and the code has been uploaded to GitHub.

**Comment 7. Expression and presentation**

Formulas are repeated and symbols are sometimes undefined. The graphics are unclear, and the abstract overly emphasizes innovation without fully explaining the results.

Operation: Equations should be numbered sequentially, with all symbols defined once and including initial conditions. Present the ODE system in a clear and consistent format and provide a schematic diagram. The abstract should explain the innovation point in one sentence and then clearly summarize the verification results. Ensure spelling and grammar are correct, and medication abbreviations are consistent (such as Met, Cap).

**Response:** These parts have been modified
