# Supplementary material for: Coupled pharmacokinetic model unveils drug-drug interactions in plasma concentration
Source: PLoS One. 2026 Feb 10;21(2):e0339052. doi: 10.1371/journal.pone.0339052 (PMC12890151; doi:10.1371/journal.pone.0339052)
Supplement: Attachment — Submitted filename: Response_to_Reviewers_auresp_2.docx [file pone.0339052.s005.docx]

**Response to Reviewers' Comments**

**Dear Editor and Reviewers,**

Hello!

Thank you for reviewing this manuscript and providing valuable feedback from the reviewers. We have carefully revised the paper based on all the review comments and explained them one by one in the response letter. The quality of the revised paper has been significantly improved.

Thank you again for your hard work and that of the reviewers.

Best wishes.

Corresponding author: Weifeng Jin; Xiaohong Li

E-mail : [jin_weifeng@126.com](mailto:jin_weifeng@126.com) (WFJ);[li_xiaoh2005@163.com](mailto:li_xiaoh2005@163.com) (XHL)

**To Academic Editor:**

**Comment 1: Linear Interaction Hypothesis**

This manuscript relies on linear coupling terms to represent drug interactions. Given that many DDIs exhibit nonlinear or saturated dynamics, a brief explanation of this hypothesis will help to enhance the credibility of physiology.

We have previously emphasized the rationale and limitations of this assumption in an earlier draft. To help readers understand and to enhance the credibility of the physiology, we have made the following changes to the limitations section in this revised draft.

The revised section is located at rows 655-670, with the changes as follows:

The model developed in this study is based on a fundamental assumption: drugs exhibit linear pharmacokinetic (PK) parameter relationships within a specific dosage range, meaning that drug-drug interactions (DDI) are assumed to follow a linear relationship. By introducing linear coupling terms, the model captures the effects between drugs. This approach simplifies the model structure and computational complexity to some extent, making it particularly suitable for scenarios with low drug concentrations where interactions primarily display approximately linear responses. However, certain drug interactions in real-world clinical settings may exhibit pronounced nonlinear or saturable pharmacokinetic characteristics. Therefore, under the assumption of solely using linear coupling terms, the model may fail to adequately characterize such nonlinear or saturable drug interactions, especially in high-dose or complex mechanism-of-action scenarios, where its predictive outcomes could deviate from reality. The introduction of the linear assumption primarily stems from a trade-off between model feasibility and interpretability, making it appropriate as an approximate framework for low-dose or relatively simple interaction conditions. For drug combinations known or suspected to involve significant nonlinear/saturable effects, it is necessary to incorporate more complex nonlinear models or real-world clinical data for supplementary analysis and correction.

**Comment 2:  Parameter identifiability**

With up to 16 parameters and limited sample sizes, the risk of parameter non-identifiability is high. The authors should comment on potential correlations among parameters and clarify how identifiability was assessed.

Our previous discussion covered the identifiability of parameters, without addressing the potential correlations between parameters.The additional content is as follows.

The revisions are located in rows 233-257, with the revised content as follows:

To effectively apply the model, accurately estimating its parameters is crucial, which must be derived precisely from experimental data. However, in the model used in this study, the number of parameters can reach up to 16, and the sample size is limited, posing risks of high parameter non-identifiability and potential parameter correlations. If all drug-related parameters are directly integrated into the modeling process, it would involve 16 parameters, resulting in nonlinear complexity and a large number of parameters. Traditional parameter determination methods may be insufficient to handle this. To address this, the parameter system is divided into two layers: the first layer consists of pharmacokinetic parameters [18] related to individual drugs, estimated independently of experimental data; the second layer comprises interaction parameters for combination therapies. The workflow of these steps is illustrated in **Fig 3**.

The parameters of a single drug can be directly obtained through experimental data analysis without relying on an optimization process, significantly reducing the dimensionality of subsequent optimization while maintaining physiological interpretability. To account for potential parameter correlations, an appropriate adjustment mechanism is designed in the fitness function during the PSO optimization process. This enables PSO to effectively intensify the optimization of highly correlated parameters, ensuring the independence and accuracy of parameter estimation. This process mitigates error amplification caused by strong correlations, further enhancing model identifiability. This hierarchical strategy not only improves the efficiency and stability of model solving but also helps clarify the hierarchical relationship between monotherapy and combination effects, strengthening the model's interpretability.

To verify the identifiability of the parameters, this study further conducted validation through bootstrap testing and ablation experiments. Bootstrap testing helps assess the model's stability across different data subsets, while ablation experiments test the model's robustness by gradually removing certain parameters or features. These methods ensure that the parameter identification results are reliable and effectively reflect the characteristics of actual drug-drug interactions.

**Comment 3: Magnitude of interaction parameters**

Several estimated interaction coefficients are extremely large relative to conventional PK rate constants. The authors should provide clarification or scaling rationale to support the biological interpretability of these values.

The revisions are located in rows 473-481, and the revision details are as follows:

It is worth mentioning that the parameters solved by the model are slightly larger compared to the results of other studies. But this is a normal phenomenon. The excessively large parameters come from **Tables 7，8**, where **Table 7** shows the parameters of each level under single drug action, and **Table 8** shows the interaction parameters under the combined action of two drugs. The reason for the large parameters comes from the dimensions and units. The time unit selected for this study is per hour (h), and the amount of medication transported from one compartment to another per hour is a cumulative process, with larger values being a normal phenomenon. This is the result of mathematical processing and does not affect the biological interpretation of the model itself.

**Comment 4: Use of individual vs. mean data**

It is unclear whether the model fitting was performed on individual-level data or aggregated means. This distinction is important for understanding variability and the robustness of the interaction estimates.

The revisions are located in rows 410-415, and the revision details are as follows:

In order for the model to more accurately describe the absorption, distribution, metabolism, and excretion processes (ADME) of drugs in different individuals, and to better capture physiological differences and dynamic changes in drug interactions between individuals, the model fitting in this study was based on individual level pharmacokinetic data, rather than aggregated mean data. If average data is used, it may lose information on individual differences, leading to distorted estimates of drug interactions in the model and affecting the final conclusion.

**Comment 5: Peripheral compartment assumptions**

Because peripheral masses are not experimentally measured, the model relies on assumed upper bounds. The authors should discuss the implications of these assumptions for parameter accuracy and model stability.

The revisions are located in rows 671-685, and the revision details are as follows:

In addition, the peripheral compartment hypothesis was also used in this study, and an upper bound was established to estimate the peripheral compartment. The upper limit of the peripheral compartment used is conservatively estimated based on existing literature and physiological knowledge, with the aim of limiting the range of drug concentrations in the peripheral compartment. The stability of the model was verified through sensitivity analysis of other key parameters and ablation experiments, and the results showed that the model has a weak dependence on the upper limit value of the peripheral compartment. In other words, within the current set range, changes in the upper limit of peripheral compartments will not significantly alter the main results of pharmacokinetics, especially the estimation of drug drug interaction effects. Therefore, this study did not conduct sensitivity analysis on the upper limit of the hypothesis of the external chamber. Although the setting of upper limits is not sensitive to the results of parameter estimation, it can still introduce errors in certain situations. In future research, it is necessary to further improve the estimation method of peripheral compartments through experimental data or advanced models (such as PBPK models) to enhance the accuracy and stability of the model and ensure more accurate prediction of drug interaction effects.

**Comment 6: Generalizability of the framework**

The proposed model is demonstrated on two drug pairs. A short discussion of how well the framework would generalize to other interaction mechanisms (e.g., transporter- or enzyme-mediated, nonlinear processes) would strengthen the manuscript’s applicability.

The revisions are located in rows 615-647, and the revision details are as follows:

Model Applicability Extension: The drug drug interaction model proposed in this study was demonstrated on two pairs of drugs, successfully demonstrating its application in pharmacokinetic analysis. However, in addition to demonstrating these two drug pairs, the model framework has strong generalizability and can be extended to handle other interaction mechanisms, especially drug interactions involving nonlinear processes mediated by transporters or enzymes.

Firstly, the absorption, distribution, metabolism, and excretion (ADME) process of drugs is typically influenced by transport proteins. For example, P-glycoprotein、 Organic anion transporters (OATP) and other transporters play a crucial role in the transmembrane transport of drugs. In this study, although the main focus is on the fundamental kinetic processes between drugs, appropriate extensions to the existing framework can be made to incorporate transporter mediated kinetic equations to describe the interactions between drugs and transporters. Especially at high drug doses, the role of transporters may exhibit saturation kinetics. To handle this nonlinear process, the Michaelis Menten dynamic model can be introduced to further enhance the adaptability of the framework. This extension not only enables the model to handle transporter mediated interactions, but also provides more accurate simulations for drug distribution and excretion processes, thereby enhancing the model's ability to estimate drug drug interaction effects.

In addition to the role of transporters, the metabolic process of drugs is also mediated by enzymes, especially the CYP450 enzyme system in the liver. The activity of these enzymes may change through drug induction or inhibition, leading to nonlinear changes in drug metabolism. In the model framework, although linear drug interactions are currently mainly dealt with, by further expanding the model, enzyme kinetics equations can be introduced to simulate the interactions between drugs and metabolic enzymes. At high concentrations, drugs may trigger enzyme saturation effects, which can be described by the Michaelis Menten kinetics of enzymes to more accurately simulate the metabolic process of drugs. Therefore, this model can adapt to drug interactions involving enzyme mediated nonlinear processes, further improving its ability to describe complex drug metabolism processes.

In the future development of the model, this framework should be further promoted to handle more complex drug drug interaction mechanisms such as immune system mediated drug interactions and the impact of gene polymorphism on drug metabolism. Meanwhile, with the accumulation of more physiological data, the model can be further refined to improve the accuracy of predicting drug interaction mechanisms. This promotion direction not only enhances the application of the model in the field of drug metabolism, but also provides theoretical support for future personalized drug treatment plans.

**We also made the necessary minor revisions.**

**1- Correct remaining pharmacological statements**

Ensure all descriptions of the imeglimin–metformin and imeglimin–cimetidine interactions explicitly reflect the findings of the cited clinical studies, which reported no clinically meaningful PK interaction. Adjust any remaining wording that implies reduced metformin concentrations, prolonged half life or enhanced imeglimin exposure.

The relevant discussion has been revised in **Table 6**, as detailed below:

Imeglimin does not significantly alter the pharmacokinetics of metformin or sitagliptin, and any observed changes in plasma concentrations are not clinically relevant [21].

2- Clarify the metoprolol–captopril description

Confirm that no text suggests captopril is metabolised by CYP2D6 or that the interaction is mediated by CYP pathways. Re-check the Results and Discussion sections to ensure consistency.

The revisions are located in rows 544-548, and the revision details are as follows:

Cap may mainly act by affecting the metabolism and excretion of Met, as suggested in reference [19]. Met is primarily metabolized in the liver, with only a small amount excreted in its original form through urine. While Cap is not a typical liver enzyme inhibitor, there is currently no evidence suggesting that Cap is metabolized by cytochrome P450 2D6, nor is the interaction between Cap and Met mediated by this enzyme [25-27].

3- Provide machine-readable data

Add CSV files containing the exact concentration–time points used for all real clinical datasets. Specify whether they were digitised or extracted from original tables.

The file 'Raw Data.csv' uploaded together is the raw data.

4- Provide code or scripts

Upload the model code (for example MATLAB, Python or equivalent) implementing:

- the coupled two-drug system,

- conversion of concentrations to mass, and

- the PSO-based optimisation with regularisation.

Update the Data Availability Statement accordingly.

The code has also been uploaded.

6- Report parameter uncertainty

Provide confidence intervals, bootstrap results or another measure of uncertainty for the estimated interaction parameters in the real clinical datasets.

The estimated values of the interaction parameters in this paper are expressed in the form of “95% CI”, and the results of the bootstrap method are shown in Table 5.

7- Add minimal diagnostic plots

Include at least one residual plot (residuals vs time or predicted vs observed) per drug in the Supplementary Materials to demonstrate that no systematic bias is present.

Attached are the plots of observed values and predicted values.

8- Clarify mapping of Drug X and Drug Y

Explicitly state, in each application, which drug is treated as X and which as Y to avoid ambiguity in the interpretation of interaction parameters.

The specific X and Y drugs have been marked on row 452.

9- Standardise terminology and simplify wording

Replace inconsistent uses of “chamber” vs “compartment”, and “mass” vs “amount”. Simplify a few long sentences in the Results and Discussion for readability.

The term 'chamber' has been standardized to 'compartment' and 'mass' has been standardized to 'amount'. Sentence structures have also been optimized.

10- Briefly address identifiability

Add a short statement in the Discussion acknowledging that some interaction parameters may be weakly informed by typical clinical sampling and that this is a recognised limitation of the approach.

Briefly mention the relevant content and row 690-691.

Additionally, some interaction parameters may be weakly influenced by typical clinical sampling, which is a recognized limitation of this method.
